# Posterior Cervical Foraminotomy Compared with Anterior Cervical Discectomy with Fusion for Cervical Radiculopathy: Two-Year Results of the FACET Randomized Noninferiority Study

**DOI:** 10.2106/JBJS.23.00775

**Published:** 2024-07-24

**Authors:** Nádia F. Simões de Souza, Anne E.H. Broekema, Michiel F. Reneman, Jan Koopmans, Henk van Santbrink, Mark P. Arts, Bachtiar Burhani, Ronald H.M.A. Bartels, Niels A. van der Gaag, Martijn H.P. Verhagen, Katalin Tamási, J. Marc C. van Dijk, Rob J.M. Groen, Remko Soer, Jos M.A. Kuijlen

**Affiliations:** 1 Department of Neurosurgery, University Medical Center Groningen, University of Groningen, Groningen, The Netherlands; 2 Department of Rehabilitation, University Medical Center Groningen, University of Groningen, Groningen, The Netherlands; 3 Department of Neurosurgery, Martini Hospital Groningen, Groningen, The Netherlands; 4 Care and Public Health Research Institute School for Public Health and Primary Care, Maastricht University, Maastricht, The Netherlands; 5 Department of Neurosurgery, Zuyderland Medical Center, Heerlen, The Netherlands; 6 Department of Neurosurgery, Maastricht University Medical Center, Maastricht, The Netherlands; 7 Department of Neurosurgery, Medical Center Haaglanden, The Hague, The Netherlands; 8 Department of Neurosurgery, Elisabeth Tweesteden Ziekenhuis, Tilburg, The Netherlands; 9 Department of Neurosurgery, Radboud University Medical Center Nijmegen, Nijmegen, The Netherlands; 10 Department of Neurosurgery, Haaglanden Medical Center, The Hague, The Netherlands; 11 Department of Neurosurgery, Haga Teaching Hospital, The Hague, The Netherlands; 12 Department of Neurosurgery, Leiden University Medical Center, Leiden, The Netherlands; 13 Department of Neurosurgery, Northwest Clinics, Alkmaar, The Netherlands; 14 Department of Epidemiology, University Medical Center Groningen, University of Groningen, Groningen, The Netherlands; 15 Department of Anesthesiology, Groninger Pain Center, University Medical Center Groningen, University of Groningen, Groningen, The Netherlands; 16 mProve Hospitals, Zwolle, The Netherlands

## Abstract

**Background:**

Posterior cervical foraminotomy (posterior surgery) is a valid alternative to anterior discectomy with fusion (anterior surgery) as a surgical treatment of cervical radiculopathy, but the quality of evidence has been limited. The purpose of this study was to compare the clinical outcome of these treatments after 2 years of follow-up. We hypothesized that posterior surgery would be noninferior to anterior surgery.

**Methods:**

This multicenter, randomized, noninferiority trial assessed patients with single-level cervical radiculopathy in 9 Dutch hospitals with a follow-up duration of 2 years. The primary outcomes measured reduction of cervical radicular pain and were the success ratio based on the Odom criteria, and arm pain and decrease in arm pain, evaluated with the visual analog scale, with a 10% noninferiority margin, which represents the maximum acceptable difference between the new treatment (posterior surgery) and the standard treatment (anterior surgery), beyond which the new treatment would be considered clinically unacceptable. The secondary outcomes were neck pain, Neck Disability Index, Work Ability Index, quality of life, complications (including reoperations), and treatment satisfaction. Generalized linear mixed effects modeling was used for analyses. The study was registered at the Overview of Medical Research in the Netherlands (OMON), formerly the Netherlands Trial Register (NTR5536).

**Results:**

From January 2016 to May 2020, 265 patients were randomized (132 to the posterior surgery group and 133 to the anterior surgery group). Among these, 25 did not have the allocated intervention; 11 of these 25 patients had symptom improvement, and the rest of the patients did not have the intervention due to various reasons. At the 2-year follow-up, of 243 patients, primary outcome data were available for 236 patients (97%). Predicted proportions of a successful outcome were 0.81 after posterior surgery and 0.74 after anterior surgery (difference in rate, −0.06 [1-sided 95% confidence interval (CI), −0.02]), indicating the noninferiority of posterior surgery. The between-group difference in arm pain was −2.7 (1-sided 95% CI, 7.4) and the between-group difference in the decrease in arm pain was 1.5 (1-sided 95% CI, 8.2), both confirming the noninferiority of posterior surgery. The secondary outcomes demonstrated small between-group differences. Serious surgery-related adverse events occurred in 9 patients (8%) who underwent posterior surgery, including 9 reoperations, and 11 patients (9%) who underwent anterior surgery, including 7 reoperations (difference in reoperation rate, −0.02 [2-sided 95% CI, −0.09 to 0.05]).

**Conclusions:**

This trial demonstrated that, after a 2-year follow-up, posterior surgery was noninferior to anterior surgery with regard to the success rate and arm pain reduction in patients with cervical radiculopathy.

**Level of Evidence:**

Therapeutic Level I. See Instructions for Authors for a complete description of levels of evidence.

Cervical radiculopathy is a common condition in the general population; the reported incidence is 83.2 per 100,000 persons per year in the general population^1^. Because it affects middle-aged to elderly individuals, often in their working phase of life, the incidence is expected to increase considerably with the world’s aging population^1,2^. Symptoms of cervical radiculopathy include pain and sensory and/or motor deficits, as a consequence of degenerative spinal nerve root compression. The associated disability can result in loss of work productivity and reduced quality of life^3,4^.

The primary treatment of cervical radiculopathy is conservative; surgery is performed when signs and/or symptoms persist. The most commonly performed surgical procedures are anterior cervical discectomy with fusion (anterior surgery) and posterior cervical foraminotomy (posterior surgery). The choice between these 2 interventions remains controversial, although many retrospective studies, and even a low-quality randomized study, have demonstrated similar clinical outcomes^5,6^. Moreover, many surgeons prefer anterior surgery, despite the potential advantages of posterior surgery (for example, fewer vital structures involved during the surgical procedure and no need for implants)^7,8^.

Given the ongoing debate, the Foraminotomy ACDF Cost-Effectiveness Trial (FACET) was designed in order to compare anterior surgery with posterior surgery. At the 1-year follow-up, the noninferiority of posterior surgery was reported^9^. This current study presents the concluding clinical and safety results after the 2-year follow-up.

## Materials and Methods

### Trial Design

The study was an investigator-blinded, multicenter, randomized, noninferiority trial performed across 9 academic and community hospitals in The Netherlands. The trial protocol was approved by the Research Ethical Board of the University Medical Center Groningen, The Netherlands. The trial design and 1-year clinical results have been published^9,10^. The Consolidated Standards of Reporting Trials (CONSORT) were followed^11^. The study was registered in the Overview of Medical Research in the Netherlands (OMON) (NTR5536).

### Trial Participants

Adult participants (18 to 80 years of age) with single-level, 1-sided cervical radiculopathy due to spondylotic neuroforaminal narrowing and/or intervertebral disc herniation, as determined by magnetic resonance imaging (MRI), that required surgical treatment were recruited. When the MRI indicated a pure spondylotic narrowing, a computed tomographic (CT) scan was performed. Patients without radicular pain, as well as those with purely axial neck pain or myelopathy (with or without radiculopathy), were not eligible. The radiographic definition of neuroforaminal narrowing and full eligibility criteria were described in the original protocol^10^. Written informed consent was obtained before randomization.

### Randomization and Blinding

Randomization was concealed and performed by research assistants at each participating site using a web-based block randomization scheme on a central computer (Trans European Network for Clinical Trials Services [TENALEA]), in a 1:1 ratio stratified by hospital. Investigators contacting patients to assess specific outcomes were blinded to the randomization sequence. Blinding of the patient and surgeon was not possible because of the nature of the surgical procedures.

### Procedures

Neurosurgeons were acquainted with both surgical techniques, which have been previously described^9,10^. Anterior surgery was performed via the standard ventral route; after discectomy with possible uncovertebral joint reduction, an intervertebral spacer (either a cage [titanium or polyetherether ketone (PEEK)] or polymethylmethacrylate [PMMA]) was applied to the disc space^12^. Posterior surgery was performed with the patient in a prone position, followed by partial hemilaminectomy and/or foraminotomy. If necessary, osteophytes and nerve-compressing disc material were removed. No additional instrumentation or braces were applied in either technique.

The trial was performed within the context of the standard level of care, including preoperative evaluation, surgical intervention, and postoperative care (including an outpatient clinic visit at 6 weeks after the surgical procedure), as per the established guidelines and practices in The Netherlands. Patients were contacted by a blinded interviewer to assess the Odom criteria and adverse events. Additionally, patients completed study questionnaires at baseline as well as at 6, 26, 52, 78, and 104 weeks postoperatively^9,10^.

### Outcomes

The centrally assessed primary outcomes measured reduction of cervical radicular pain and included the proportion of patients with success (“excellent” or “good”) on the 4-item Odom scale and postoperative arm pain assessed with a visual analog scale (VAS) for self-reported arm pain (0 to 100), in which a lower score and a greater decrease from baseline indicate a greater decrease in pain^13^.

The secondary outcomes consisted of multiple Dutch validated questionnaires, including the VAS neck pain score, Neck Disability Index (10 items, 0 to 50; the sum of the scores is doubled to yield a percentage), EuroQol-5 Dimensions-5 Level questionnaire (EQ-5D-5L), and Work Ability Index Single Item^14-17^. Although not explicitly mentioned in the registry study information, treatment satisfaction (rated on a 1 to 7-item scale) was included as a secondary outcome in the trial, as this outcome reflects participants’ subjective experiences with regard to the intervention that they underwent, and we decided to include it because of its clinical relevance. Finally, our registry listed questionnaires regarding productivity-related costs and medical costs, but we did not include them in the current study because they will be discussed in an upcoming study on cost-effectiveness.

The thresholds for minimal clinically important differences (MCIDs) for the Neck Disability Index, EQ-5D-5L, and VAS arm and neck pain scores were reported in the study protocol^10^. Complications (including reoperations and adverse events [i.e., any unexpected medical occurrence without direct causal relation to the studied treatments]) were documented. Adverse events were regarded as serious if they were lethal or life-threatening, required prolongation of hospitalization, or caused substantial disability or any other medically important event jeopardizing the subject or requiring intervention.

### Statistical Analysis

Details of the analytic approach and power calculation have been published previously, and the complete statistical analysis plan is available with the protocol^9,10^. The sample size of the study was calculated on the basis of the Odom criteria, chosen because of their wide adoption in spine surgery and sufficient measurement properties^18,19^. The proportion of patients with a successful outcome was hypothesized to be similar after anterior and posterior surgery; therefore, an overall success rate of 87% was assumed on the basis of the largest available review in the literature^18^. A noninferiority margin of 10% was chosen in light of potential advantages of posterior surgery, such as avoiding fusion-related complications and preserving postoperative range of motion as well as potentially lower costs that would justify a tolerable loss of efficacy of 10%. Assuming a 1-sided alpha of 0.05, power of 80%, and 10% loss to follow-up, the targeted sample size to rule out a between-group difference in the rate of a successful outcome was calculated to be 308 patients. Unfortunately, there was a lower inclusion rate than anticipated, partly due to the COVID-19 pandemic and its associated cancellation of non-emergency health care. An interim analysis and power calculation performed by a statistician not involved in the study design indicated that it was safe to end the inclusion at 86% of the predefined sample size with low risks of false-negatives^9^. Details on post hoc power calculations including all data after 2 years of follow-up for both primary outcomes are presented in Appendix Table S1. Analyses of primary and secondary end points were based on the intention-to-treat principle.

The noninferiority of primary outcomes (Odom criteria and VAS arm pain) was tested at a 1-sided 95% confidence interval (CI) with a noninferiority margin of 10%, with a Bonferroni correction for multiple testing. Unadjusted between-group differences for VAS arm pain and the decrease in VAS arm pain were calculated with linear mixed effects modeling, including the baseline score and treatment group as independent variables. Since between-group differences could vary among follow-up times, time was included as categorical variable. A time-by-treatment interaction was included if it resulted in an improved model fit. A random intercept per patient was included. The 4-point Odom score was dichotomized, and logistic mixed effects modeling was then performed according to the abovementioned specifications, except that time was included as a continuous variable and patients varying over time were included as a random effect. Subsequently, bootstrapping (n = 1,000) was performed to obtain 95% CIs on a proportional scale. Adjusted between-group differences were calculated by including potential confounders as independent variables. These confounders were sex, age, body mass index (BMI), smoking, and use of pain medication. In addition, sensitivity analyses, including per-protocol, complete case, and multiple imputation analyses, were performed to test the robustness of the primary outcomes.

All secondary outcomes were analyzed exploratively using 2-sided 95% CIs, as no noninferiority margin was specified for the secondary outcomes. Responder analyses were performed for outcomes with a known MCID threshold^10,20^. Adverse events and reoperations were reported descriptively. All analyses were performed in R (version 4.0.5; The R Foundation).

## Results

### Trial Participants

**Fig. 1:**
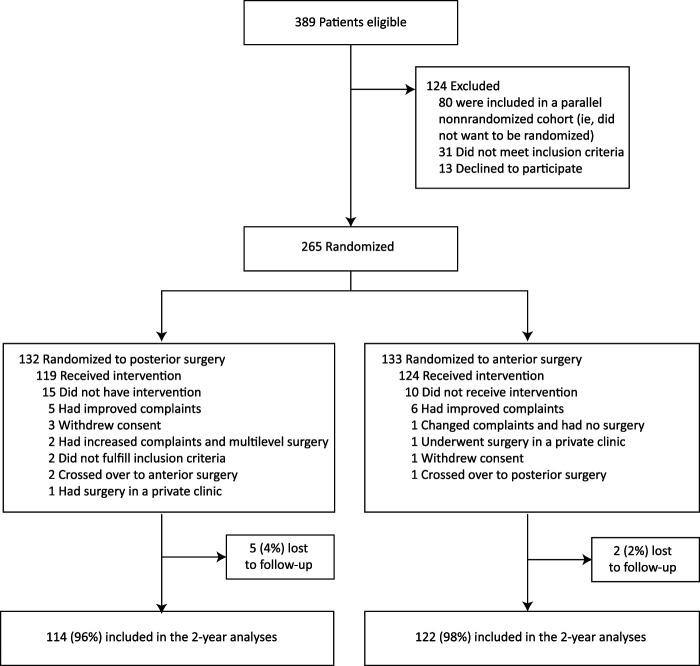
Flowchart of patient randomization, primary treatment, and follow-up status. Participants who were included in the 2-year analyses of the primary outcomes had available data for the Odom score and/or the VAS for arm pain, as prespecified in the study protocol. (Reproduced, with permission from JAMA Neurology. Noninferiority of Posterior Cervical Foraminotomy vs Anterior Cervical Discectomy With Fusion for Procedural Success and Reduction in Arm Pain Among Patients With Cervical Radiculopathy at 1 Year: The FACET Randomized Clinical Trial. 2023. 80[1]:40-8. Copyright © 2022 American Medical Association. All rights reserved, including those for text and data mining, AI training, and similar technologies.)

From January 2016 through May 2020, eligibility was assessed in 389 patients; 31 did not meet inclusion criteria, 13 declined to participate, and 80 did not want to be randomized. The remaining 265 patients were randomized. The final group allocation included 132 patients randomized to posterior surgery and 133 patients randomized to anterior surgery. After randomization, 25 patients did not have the allocated intervention, mostly because of spontaneous improvement of symptoms, leaving 119 patients in the posterior surgery group and 124 patients in the anterior surgery group in the intention-to-treat analysis (including 3 crossovers) (Fig. 1). As prespecified in the study protocol, patients were considered lost to follow-up when neither the Odom score nor the VAS arm pain score was available at the final follow-up. As such, 5 patients (4%) in the posterior surgery group and 2 patients (2%) in the anterior surgery group were considered lost to follow-up at 2 years. The detailed characteristics of the included patients have been previously published^9^. Although we tested for significance in differences between groups and did not find any significant differences, p values were not reported (Table I).

**TABLE I:** Baseline Characteristics of Included Patients*

Characteristic	Posterior Surgery (N = 119)	Anterior Surgery (N = 124)
Age† *(yr)*	51.6 ± 8.5	51.0 ± 8.3
Sex‡		
Female	66 (55%)	58 (47%)
Male	53 (45%)	66 (53%)
BMI§ *(kg/m*^*2*^*)*	27 (24 to 30)	27 (24 to 30)
Dermatome clinical diagnosis‡		
C5, right	1 (1%)	1 (1%)
C5, left	1 (1%)	0 (0%)
C6, right	29 (24%)	37 (30%)
C6, left	30 (25%)	24 (19%)
C7, right	21 (18%)	26 (21%)
C7, left	37 (31%)	36 (29%)
Symptom duration§ *(wk)*	34 (26 to 52)	32 (20 to 52)
ASA classification‡#		
I	55 (46%)	66 (53%)
II	59 (50%)	53 (43%)
III	5 (4%)	5 (4%)
Current smoker‡**	53 (46%)	47 (39%)
Use of nonsteroidal anti-inflammatory drugs‡††	39 (33%)	35 (29%)
Radiographic characteristics‡,‡‡		
Discogenic (soft disc)	48 (40%)	38 (31%)
Spondylotic	14 (12%)	14 (11%)
Combined discogenic and spondylotic	57 (48%)	70 (57%)
Clinical characteristics‡		
Radiating arm and neck pain	56 (47%)	61 (49%)
Radiating arm pain only	63 (53%)	63 (51%)
Loss of strength	43 (36%)	51 (41%)
Loss of sensibility	79 (66%)	82 (66%)
Tingling in fingers or hand	93 (78%)	106 (85%)
Comorbidities	66 (55%)	57 (46%)
Specific comorbidities§§		
Cardiovascular	36	26
Pulmonary	22	20
Endocrine	19	18
Musculoskeletal	16	14
Gastrointestinal and liver	7	10
Neurological	3	10
Thromboembolic	5	4
Psychiatric	2	2
Oncological	4	1
Nephrological	2	1
Clinically relevant other	2	4

* Reproduced with permission from JAMA Neurology, Noninferiority of Posterior Cervical Foraminotomy vs Anterior Cervical Discectomy With Fusion for Procedural Success and Reduction in Arm Pain Among Patients With Cervical Radiculopathy at 1 Year: The FACET Randomized Clinical Trial. 2023 Jan 1. 80(1):40-8. Copyright © 2023 American Medical Association. All rights reserved, including those for text and datamining, AI training, and similar technologies. ASA = American Society of Anesthesiologists.

† The values are given as the mean and the standard deviation.

‡ The values are given as the number of patients, with the percentage in parentheses; percentages may not total 100 because of rounding.

§ The values are given as the median, with the interquartile range in parentheses.

# The ASA classification system ranges from I to VI, where higher classes indicate a greater risk; no patients had an ASA IV, V, or VI classification.

** Data were missing for 3 patients in the posterior surgery group and 2 patients in the anterior surgery group.

†† Data were missing for 1 patient in the posterior surgery group and 2 patients in the anterior surgery group.

‡‡ Data were missing for 2 patients in the anterior surgery group.

§§ The values are given as the number of patients. The specification of comorbidities is at the event level, not the patient level; therefore, no percentages are given, because several patients had multiple comorbidities.

### Primary Outcomes

**Fig. 2:**
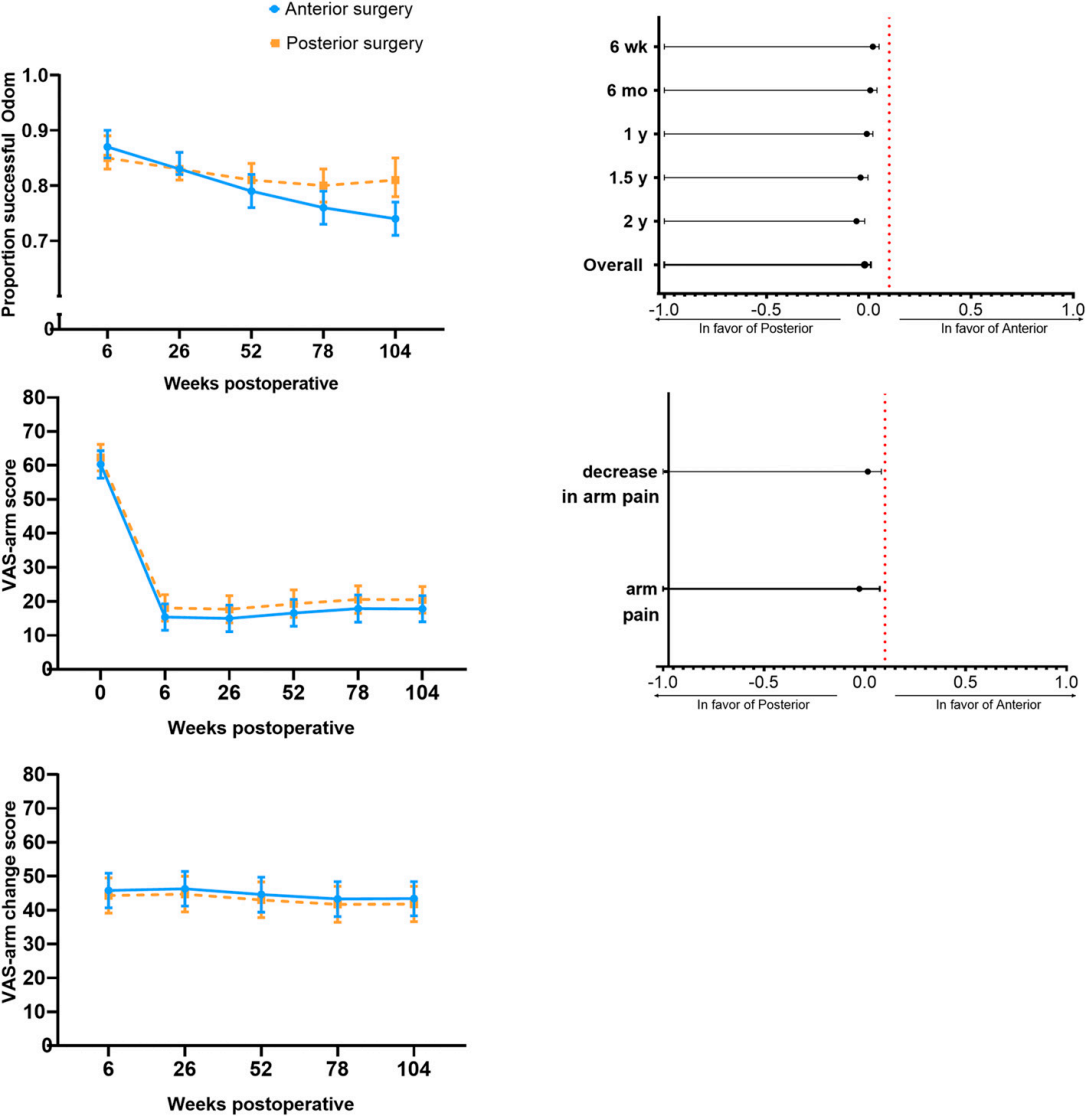
Results for the primary outcomes. The error bars in the top left, middle left, and bottom left indicate the 2-sided 95% CI. *Top left:* The model-based estimated proportions of patients with a successful outcome (per the Odom criteria) are depicted for the posterior and anterior surgery groups. The Odom score was not measured at baseline. *Middle left:* The observed baseline scores and subsequent model-based estimated mean VAS arm pain scores are given for the posterior and anterior surgery groups. *Bottom left:* The model-based estimated changes in VAS arm pain from baseline to the follow-up time points are given for posterior and anterior surgery. No baseline score is given for the VAS arm pain score, as the VAS arm pain change score is calculated by subtracting follow-up scores from baseline. *Top right:* The model-based point estimates, with accompanying 1-sided 95% CIs, for the difference between anterior surgery and posterior surgery are given. Noninferiority was established at each follow-up time point. The red dotted line denotes the noninferiority margin of 0.1. *Middle right:* The model-based point estimates, with accompanying 95% CIs, are given for the between-group difference in mean VAS arm pain and for the decrease in mean VAS arm pain from baseline. The red dotted line denotes the noninferiority margin of 0.1.

At the 2-year follow-up, primary outcome data were available for 236 patients (97% of 243). After 2 years, the proportion of patients with a successful outcome was 0.81 in the posterior surgery group and 0.74 in the anterior surgery group (difference, −0.06 [1-sided 95% CI, −0.02]), indicating noninferiority of posterior surgery (and as the 1-sided 95% CI did not encompass 0, superiority was also demonstrated for this particular outcome measure) (Fig. 2). Overall and between-group differences at each follow-up time point demonstrated the noninferiority of posterior surgery (Fig. 2; see also Appendix Table S2).

The mean arm pain score decreased from 62.3 (95% CI, 58.4 to 66.2) at baseline to 20.5 (95% CI, 16.6 to 24.4) at 2 years in the posterior surgery group. In the anterior surgery group, a decrease from 60.3 (95% CI, 56.2 to 64.3) to 17.8 (95% CI, 14.0 to 21.7) was noted. Overall, the between-group difference in mean arm pain was −2.7 (1-sided 95% CI, 7.4), which was stable over time and confirmed the noninferiority of posterior surgery. Overall, the between-group difference for the decrease in arm pain was 1.5 (1-sided 95% CI, 8.2), also supporting the noninferiority of posterior surgery (Fig. 2; see also Appendix Table S2). The adjusted between-group differences and sensitivity analyses for both primary outcomes yielded similar results, except for the decrease in arm pain scores in complete cases (1-sided 95% CI, 10.6). The results of the adjusted analysis and sensitivity analyses are presented in Appendix Tables S3, S4, and S5.

### Secondary Outcomes

Small between-group differences were observed for model-based mean neck pain, disability, work ability, quality of life, and treatment satisfaction, with 2-sided 95% CIs including zero (Table II). All outcomes achieved the majority of improvement by 6 months in both groups and remained relatively stable thereafter. Additionally, the mean change scores for all outcomes except the EQ-5D-5L reached the predefined MCID threshold (see Appendix Table S6). The proportions of responders who reached the MCID for arm pain did not change over time; after 2 years, proportions were 0.56 in the posterior surgery group and 0.58 in the anterior surgery group (difference, 0.02 [2-sided 95% CI, −0.05 to 0.05]) (see Appendix Table S7).

**TABLE II:** Secondary Outcome Measures*

Outcome	Posterior Surgery (N = 119)			Anterior Surgery (N = 124)			Anterior vs. Posterior Difference†
	Observed	Predicted	95% CI	Observed	Predicted	95% CI	
Mean VAS neck pain							
Baseline	55.2	55.2	51.0 to 59.4	53.6	53.6	49.3 to 58.0	—
6 weeks‡	25.2	25.3	21.0 to 29.6	23.0	25.2	21.0 to 29.4	−0.09 (−5.2 to 5.0)
6 months§	20.8	22.6	18.3 to 27.0	22.7	22.5	18.2 to 26.8	−0.09 (−5.2 to 5.0)
1 year#	24.4	24.6	20.3 to 29.0	21.7	24.5	20.2 to 28.8	−0.09 (−5.2 to 5.0)
1.5 years**	24.5	26.9	22.4 to 31.3	24.6	26.8	22.4 to 31.2	−0.09 (−5.2 to 5.0)
2 years††	23.0	25.7	21.4 to 30.0	25.1	25.6	21.4 to 29.8	−0.09 (−5.2 to 5.0)
Mean Neck Disability Index							
Baseline	43.6	43.6	40.9 to 46.1	42.2	42.2	39.8 to 44.7	—
6 weeks‡	26.7	24.5	22.0 to 27.1	23.9	25.5	23.0 to 28.0	0.9 (−2.3 to 4.2)
6 months§	18.9	18.2	15.6 to 20.8	19.9	19.2	16.6 to 21.7	0.9 (−2.3 to 4.2)
1 year#	17.6	18.0	15.3 to 20.6	19.2	18.9	16.4 to 21.5	0.9 (−2.3 to 4.2)
1.5 years**	18.3	18.7	16.1 to 21.4	19.2	19.7	17.1 to 22.3	0.9 (−2.3 to 4.2)
2 years††	18.5	19.3	16.7 to 21.8	21.0	20.2	17.7 to 22.7	0.9 (−2.3 to 4.2)
Mean Work Ability Index Single Item							
Baseline	3.8	3.8	3.3 to 4.3	3.8	3.8	3.3 to 4.3	—
6 weeks‡	5.0	5.0	4.6 to 5.5	5.5	5.6	5.1 to 6.0	0.5 (−0.1 to 1.2)
6 months§	6.5	6.4	5.9 to 6.9	6.6	6.8	6.3 to 7.3	0.4 (−0.3 to 1.0)
1 year#	6.7	6.7	6.2 to 7.1	6.6	6.7	6.2 to 7.1	−0.02 (−0.7 to 0.6)
1.5 years**	6.9	6.9	6.4 to 7.4	6.6	6.6	6.1 to 7.0	−0.3 (−1.0 to 0.3)
2 years††	6.7	6.6	6.2 to 7.1	6.4	6.4	5.9 to 6.8	−0.3 (−0.9 to 0.4)
Mean EQ-5D-5L							
Baseline	0.61	0.61	0.57 to 0.64	0.62	0.62	0.58 to 0.65	—
6 weeks‡	0.77	0.78	0.76 to 0.81	0.79	0.77	0.75 to 0.80	−0.01 (−0.04 to 0.02)
6 months§	0.81	0.82	0.79 to 0.84	0.81	0.81	0.78 to 0.83	−0.01 (−0.04 to 0.02)
1 year#	0.84	0.83	0.80 to 0.86	0.82	0.82	0.79 to 0.84	−0.01 (−0.04 to 0.02)
1.5 years**	0.83	0.82	0.79 to 0.85	0.81	0.81	0.78 to 0.84	−0.01 (−0.04 to 0.02)
2 years††	0.82	0.81	0.78 to 0.84	0.80	0.80	0.77 to 0.82	−0.01 (−0.04 to 0.02)
Satisfaction score‡‡§§							
6 weeks‡	0.85	0.87	0.85 to 0.90	0.88	0.86	0.85 to 0.90	−0.004 (−0.03 to 0.03)
6 months§	0.87	0.86	0.85 to 0.89	0.85	0.85	0.85 to 0.89	−0.004 (−0.03 to 0.03)
1 year#	0.85	0.86	0.84 to 0.88	0.85	0.85	0.83 to 0.89	−0.004 (−0.03 to 0.03)
1.5 years**	0.87	0.85	0.83 to 0.88	0.84	0.84	0.83 to 0.88	−0.004 (−0.03 to 0.03)
2 years††	0.84	0.85	0.82 to 0.88	0.85	0.84	0.82 to 0.88	−0.004 (−0.03 to 0.03)
All adverse events##***							−0.06 (−0.2 to 0.07)
1	37 (31%)	—	—	—	28 (23%)	—	−0.09 (−0.2 to 0.03)
>1	8 (7%)	—	—	—	13 (10%)	—	0.04 (−0.04 to 0.1)
Total	45 (38%)	—	—	—	41 (33%)	—	−0.05 (−0.2 to 0.08)
Surgery-related adverse events##							
1	25 (21%)	—	—	—	21 (17%)	—	−0.04 (−0.1 to 0.07)
>1	3 (3%)	—	—	—	4 (3%)	—	0.007 (−0.04 to 0.06)
Total	28 (24%)	—	—	—	25 (20%)	—	−0.03 (−0.1 to 0.08)
All serious adverse events##***							
1	13 (11%)	—	—	—	23 (19%)	—	0.08 (−0.02 to 0.2)
>1	2 (2%)	—	—	—	1 (1%)	—	−0.009 (−0.04 to 0.03)
Total	15 (13%)	—	—	—	24 (19%)	—	0.07 (−0.03 to 0.2)
Surgery-related serious adverse events##***							
1	7 (6%)	—	—	—	11 (9%)	—	0.03 (−0.04 to 0.1)
>1	2 (2%)	—	—	—	0 (0%)	—	−0.02 (−0.05 to 0.02)
Total	9 (8%)	—	—	—	11 (9%)	—	0.01 (−0.06 to 0.09)
Reoperations##	9 (8%)	—	—	—	7 (6%)	—	−0.02 (−0.09 to 0.05)

* Baseline values are observed data, and unadjusted follow-up values are estimated by generalized linear mixed models including baseline score, intervention groups, and time as categorical variables. A time-by-treatment interaction was included if it yielded improved model fit, tested by forward selection with maximum likelihood estimation. The predicted means and between-group differences with 2-sided 95% CIs are given for follow-up measurements, except for the satisfaction score, serious adverse events, and reoperations. For these outcomes, the reported values denote proportions with 2-sided 95% CIs (satisfaction score) or the number of patients with percentages in parentheses (serious adverse events and reoperations).

† The values are given as the difference between groups, with the 95% CI in parentheses.

‡ At 6 weeks, data were missing for 17 patients in the anterior surgery group and 16 patients in the posterior surgery group.

§ At 6 months, data were missing for 22 patients in the anterior surgery group and 20 patients in the posterior surgery group.

# At 1 year, data were missing for 25 patients in the anterior surgery group and 23 patients in the posterior surgery group.

** At 1.5 years, data were missing for 33 patients in the anterior surgery group and 29 patients in the posterior group.

†† At 2 years, data were missing for 14 patients in the anterior surgery group and 12 patients in the posterior surgery group.

‡‡ In a questionnaire on overall satisfaction, patients responded to the following question: “How satisfied are you with the results of the surgery?” The 7-point answer options were “very satisfied,” “satisfied,” “moderately satisfied,” “somewhat satisfied/not satisfied,” “slightly dissatisfied,” “dissatisfied,” and “very dissatisfied.” The number of patients who answered with “very satisfied,” “satisfied,” or “moderately satisfied” is given. It was not possible to bootstrap the proportions with their 95% CIs using a model with random effects; therefore, the same model without random effects was used in the bootstrapping, and the results are presented here. The proportions in the models with and without random effects differed by ≤2.7%.

§§ Bootstrapping was used to calculate the 95% CIs because the model output for satisfaction score (a binarized outcome) was in log odds. As a result, the bootstrapped CIs do not exactly match the estimates, with all estimates being near one edge of the CI.

## The values are given as the number of patients, with the percentage in parentheses.

*** Adverse events were considered serious if they were lethal or life-threatening, required prolonged hospitalization, caused substantial disability, were a congenital anomaly or birth defect, or were any other medically important event that jeopardized the subject or required intervention. All of the serious adverse events were calculated at the patient level. For an overview of the adverse event levels, see Appendix Table S8.

#### Safety Outcomes

Serious surgery-related adverse events occurred in 9 patients (8%) in the posterior surgery group and 11 patients (9%) in the anterior surgery group; 9 patients in the posterior group and 7 patients in the anterior group had a revision surgical procedure within 2 years (difference in reoperation rate, −0.02 [2-sided 95% CI, −0.09 to 0.05]) (Table II; see also Appendix Table S8). Reoperations were performed at the index level only in 7 of the 9 patients who underwent posterior surgery and 3 of the 7 patients who underwent anterior surgery; at the index level and the adjacent level in 2 of the 9 patients who underwent posterior surgery; at the adjacent level only in 3 of the 7 patients who underwent anterior surgery; and at a different level in 1 of 7 patients who underwent anterior surgery (Table III; see also Appendix Table S9). Adverse events related to the surgical procedure occurred in 28 patients (24%) who underwent posterior surgery and 25 patients (20%) who underwent anterior surgery, with the largest between-group difference being for recurrent radicular symptoms without reoperation, in 8 patients (7%) who underwent posterior surgery and 1 patient (1%) who underwent anterior surgery (difference in rate, −0.06 [2-sided 95% CI, −0.1 to −0.003]) (see Appendix Table S10). All recurrences were referred to a pain specialist; 5 of 8 patients with recurrences underwent a selective nerve blockage.

**TABLE III:** Reoperations Within 2 Years of Follow-up

Patient	Initial Treatment	Initial Symptomatic Nerve Root	Reoperation	Symptomatic Nerve Root at Reoperation	Reason for Reoperation	Months After Initial Surgery
Posterior surgery group						
1	Posterior surgery at C6	C6 right	Anterior surgery at C5-6	C6 right	Recurrent symptoms	1
2	Posterior surgery at C6	C6 right	Anterior surgery at C5-6	C6 right	Recurrent symptoms	4
3	Posterior surgery at C7	C7 left	Anterior surgery at C5-6 and C6-7	C6 right and C7 left	Recurrent and new symptoms*	6
4	Posterior surgery at C7	C7 left	Anterior surgery at C5-6 and C6-7	C6 bilateral and C7 left	Recurrent and new symptoms†	10
5	Posterior surgery at C6	C6 right	Anterior surgery at C5-6	C6 right	Recurrent symptoms‡	10
6	Posterior surgery at C6	C6 left	Anterior surgery at C5-6	C6 right	New symptoms, right	12
7	Posterior surgery at C6	C6 left	Anterior surgery at C5-6	C6 left	Recurrent symptoms	13
8	Posterior surgery at C7	C7 left	Anterior surgery at C6-7	C7 left	Recurrent symptoms	19
9	Posterior surgery at C6	C6 left	Anterior surgery at C5-6	C6 left	Recurrent symptoms	23
Anterior surgery group						
1	Anterior surgery at C6-7	C7 right	Anterior surgery at C5-6	C6 right	New symptoms, right	2
2	Anterior surgery at C5-6	C6 right	Anterior surgery at C5-6	C6 right	Removal intervertebral cage§	7
3	Anterior surgery at C5-6	C6 right	Anterior surgery at C6-7	C7 right	New symptoms, right	8
4	Anterior surgery at C5-6	C6 right	Posterior surgery at C6	C6 right	Recurrent symptoms	10
5	Anterior surgery at C6-7	C7 left	Anterior surgery at C6-7	C7 left	Recurrent symptoms#	21
6	Anterior surgery at C5-6	C6 right	Posterior surgery at C8	C8 right	New symptoms, right	24
7	Anterior surgery at C5-6	C6 left	Posterior surgery at C7	C7 left	New symptoms, left	24

* This patient had a reoperation for recurrent symptoms at the left C7 nerve root with additional new symptoms due to a herniated disc at the right C5-6 level.

† This patient had a reoperation because of recurrent symptoms at the left C7 nerve root with increased disc protrusion on imaging in combination with a bilateral herniated disc at the C5-6 level.

‡ This patient needed another surgery for the wound drain to be removed.

§ This patient had the cage removed because of malpositioning and a low-grade infection.

# This patient was treated with reoperation for persistent foraminal stenosis of the left C7 level confirmed on cervical imaging. This patient had the intervertebral spacer removed, removal of remaining osteophytes, and placement of a cage.

## Discussion

The 2-year longitudinal results of this multicenter randomized trial that treated patients with single-level, unilateral, cervical radiculopathy demonstrated noninferiority of posterior surgery compared with anterior surgery, in terms of the success ratio and arm pain. The secondary and safety outcomes showed nonsignificant differences between groups. These results are consistent with the current literature, in which similar clinical outcomes have been demonstrated after posterior surgery and anterior surgery, with follow-up duration ranging from 1 to 72 months^5,9^. Because the current literature of which we are aware consists of mainly retrospective studies, our findings provide Level-I evidence that posterior surgery is noninferior to anterior surgery with regard to the clinical outcome, with follow-up of 2 years.

After 2 years, 9 patients (8%) in the posterior surgery group and 7 patients (6%) in the anterior surgery group underwent a reoperation, which is slightly higher than we observed after the 1-year follow-up (5% in the posterior surgery group compared with 3% in the anterior surgery group)^9^. The between-group difference of 2% is smaller than in a previous meta-analysis reporting a 4.5% difference between open posterior surgery and anterior surgery, which did not reach significance (p = 0.06)^5^. However, it should be noted that this meta-analysis also provided a subanalysis showing a nonsignificant difference between minimally invasive posterior surgery and anterior surgery (p = 0.18) along with a pooled estimate (p = 0.02) favoring anterior surgery. The significance of this pooled estimate is likely influenced by only 1 retrospective study included in the subanalysis comparing minimally invasive posterior surgery with anterior surgery, which consisted of 55 patients in the anterior surgery group and 21 patients in the minimally invasive posterior surgery group, demonstrating a clear significant difference favoring anterior surgery. Another meta-analysis comparing only minimally invasive posterior surgery with anterior surgery did not find a significant difference in reoperation rates^21^. Studies of higher quality that include more patients should be conducted to permit drawing better conclusions regarding whether minimally invasive posterior surgery is worse in terms of reoperations compared with open posterior surgery or anterior surgery^5,21^.

Moreover, we demonstrated that all reoperations after posterior surgery were at the index level (the level at which the surgeons originally performed the operation; 2 patients in the posterior surgery group underwent reoperation at the index level and the adjacent level) because of persistent or reappearing radicular pain, whereas reoperations after anterior surgery were mostly (43%) aimed at adjacent levels only or at a non-adjacent different level (14%). More reoperations at adjacent levels after anterior surgery could be attributed to the development of adjacent segment disease, because of decreased motion at the index level resulting in hypothetical overstraining of the adjacent level. There is conflicting evidence on this matter, as case series with long follow-up have shown rates of adjacent segment disease varying between 0.6% and 2.9% after anterior surgery^22-24^. Additionally, reoperations at the index level after posterior surgery could be attributed to the indirect decompression when a herniated disc is not removed. It would be interesting to study the reoperation rates in our patients after a longer follow-up.

As previously described, all adverse events were conscientiously documented and assessed in terms of their relation to the surgery^9^. The rates of surgery-related serious adverse events after 2 years did not differ between posterior surgery and anterior surgery. However, as expected, the types of complications were slightly different between groups, with the largest difference being recurrent symptoms without reoperation, occurring in 8 patients (7%) in the posterior surgery group and 1 patient (1%) in the anterior surgery group (difference in rate, −0.06 [95% CI, −0.1 to −0.003]). It remains difficult to identify the exact reason for this between-group difference. Theories include manipulation of the nerve root during surgery or indirect decompression if a herniated disc is not removed. Another hypothesis is that the nature of the nerve root compression (spondylotic compared with soft disc) may play a role, although opinions differ among the surgeons in our study.

Finally, all secondary outcomes improved after surgery in both groups, including neck pain. Our observed improvement of neck pain after both surgeries is in line with previous literature, although our between-group difference is smaller compared with previous literature^25,26^. Moreover, the majority of the neck pain improvement occurred by 6 months, and remained relatively stable thereafter. We previously published the short-term neck pain results after posterior surgery compared with anterior surgery and demonstrated that neck pain scores were initially higher in the posterior surgery group in the first 4 weeks, but, thereafter, the results in the 2 surgery groups were similar^27^. The initially higher neck pain after posterior surgery was attributed to the nature of the posterior approach, in which muscles in the neck are retracted during the surgical procedure and need to recover. However, the mechanism of neck pain improvement from 6 weeks to 6 months after posterior or anterior surgery remains poorly understood.

The clinical effectiveness results after 2 years of follow-up, including safety measures, are relevant to all patients with cervical radiculopathy and to a wide range of specialists involved in their treatment. Moreover, given the current existing preference for anterior surgery, widespread awareness of these results is important, as both procedures should be discussed in patient counseling. We robustly demonstrated posterior surgery to be noninferior to anterior surgery, with similar primary and secondary outcomes after 2 years of follow-up. Posterior surgery has advantages over anterior surgery, such as avoidance of vital structures, no need for an intervertebral spacer, a better economic profile, and maintenance of range of motion (although disc arthroplasty could also be considered as a motion-sparing technique)^28,29^. However, it also includes drawbacks such as more neck pain in the short term^27^. Moreover, although the study was not powered to draw firm conclusions regarding adverse events, we found slightly higher rates of reoperation and recurrent symptoms after posterior surgery. As both procedures have similar clinical outcome profiles, the emphasis in patient counseling should be on the types of complications for each procedure, patient-specific factors, and potential sustainability (fewer costs for posterior surgery). Both the physician and patient should individually weigh the advantages and disadvantages of both procedures.

A limitation of this study was that the predefined sample size was not reached. However, interim analysis proved that it was safe to end the study, with low risks of false-negatives. Also, the power of the study was calculated on the basis of the Odom score only, whereas noninferiority was tested for both primary outcomes. However, longitudinal post hoc power calculations for both primary outcomes showed adequate power, and sensitivity analyses demonstrated robustness of both noninferiority results. Other important limitations were the inability to blind surgeons and patients to the intervention and the existing preference among surgeons for anterior surgery, which could have led to selection bias. Also, we did not routinely perform CT scans preoperatively, only in cases with suspected isolated spondylotic changes. Finally, an evidence-based noninferiority margin did not exist; the 10% margin was therefore chosen empirically.

In conclusion, this randomized controlled trial demonstrated consistent noninferiority of posterior surgery compared with anterior surgery with regard to the success rate and arm pain at the 2-year follow-up in patients with cervical foraminal radiculopathy. All secondary outcomes showed similar results between groups. The rates of serious adverse events were comparable, whereas the types of complications differed between groups.

## Appendix

Supporting material provided by the authors is posted with the online version of this article as a data supplement at jbjs.org (http://links.lww.com/JBJS/I108).

## Data Availability

A **data-sharing statement** is provided with the online version of the article (http://links.lww.com/JBJS/I109).
